# Emotion Regulation Flexibility: Gender Differences in Context Sensitivity and Repertoire

**DOI:** 10.3389/fpsyg.2019.00935

**Published:** 2019-05-09

**Authors:** K. Elise Goubet, Evangelia G. Chrysikou

**Affiliations:** ^1^Department of Psychology, The University of Kansas, Lawrence, KS, United States; ^2^Department of Psychology, Drexel University, Philadelphia, PA, United States

**Keywords:** emotion regulation flexibility, context sensitivity, repertoire, gender differences, emotion regulation

## Abstract

Emotion regulation (ER) has been conceptualized as processes through which individuals modulate their emotions consciously and non-consciously to respond appropriately to environmental demands. Emotions can be regulated in many ways and specific strategies may have differing efficacy across situations and individuals. The importance of flexibility in implementing ER strategies has been highlighted in many current models. In this study, we investigated gender differences in two regulatory processes, context sensitivity and repertoire using a novel coding system for ER strategy classification. The results revealed that women consistently used more strategies than men and were more flexible in the implementation of those strategies. These findings validate our novel coding system for ER strategy classification. They further highlight the importance of a comprehensive examination of gender differences in ER processes for understanding the nuances of ER and developing effective treatments for psychopathologies characterized by ER deficits.

## Introduction

Everyday life is filled with attempts to change the way we feel. A teenager goes off on an eating binge when she feels lonely or depressed. A student frets over a failed exam, thinking over and over about what he could have done differently. A young woman, devastated by her partner’s cheating, leans on friends for comfort and advice. These are all examples of emotion regulation (ER): ‘the set of automatic and controlled processes involved in the initiation, maintenance, and modification of the occurrence, intensity, and duration of feeling states’ (Gross and Thompson, 2007). Successful ER has been associated with overall well-being and psychological health (Gross and John, 2003). On the other hand, deficits in ER have been implicated in an estimated 40–75% of different psychopathologies, including mood and anxiety disorders (see Aldao et al., 2010; Gross and Jazaieri, 2014; Joormann and Stanton, 2016). A characteristic of psychopathologies marked by ER deficits is the presence of significant gender differences in the prevalence of these disorders (for a review see Nolen-Hoeksema, 2012). Although these gender effects have not been thoroughly explored, one hypothesis suggests that gender differences in ER may, in part, explain the gender differences in the clinical presentation of these psychopathologies (Hyde et al., 2008; Nolen-Hoeksema, 2012).

In line with this prediction, many ER processes have been characterized by significant gender effects, including the frequency of employment of specific ER strategies (e.g., Nolen-Hoeksema and Jackson, 2001; Tamres et al., 2002; Nolen-Hoeksema and Aldao, 2011; Kwon et al., 2013) or the extent of involvement of different neural systems associated with ER (e.g., Koch et al., 2007; McRae et al., 2008; Domes et al., 2010; Whittle et al., 2011). Nevertheless, in most of the research exploring ER with functional neuroimaging, gender is either held constant or not adequately examined because of unequal or small sample sizes. As such, although gender effects likely influence the interpretation and generalizability of the results of these studies within ER research (for a review see Cahill, 2006; Koch et al., 2007), they remain largely unexplored.

Gender differences in ER might be particularly prevalent in the context of flexible ER choice. ER flexibility is thought to comprise three components: sensitivity to situational demands (context), availability of a diverse array of strategies (repertoire), and the ability to switch strategies if needed (responsiveness to feedback; Bonanno and Burton, 2013). More than a decade of research has shown that emotions can be regulated in many ways and that the application of ER strategies can have different consequences in different situations (Webb et al., 2012). Conceptual accounts suggest that flexible choice between ER strategies is central to well-being and that various forms of psychopathology can be characterized by a breakdown in this flexibility (Kashdan and Rottenberg, 2010). For this reason, current theoretical models of ER are pulling away from the common assumption that there are “good” or “bad,” “adaptive” or “maladaptive” ER strategies, proposing instead that successful ER is not determined simply by the types of strategies used, but rather by the flexibility in their application depending on context (for reviews, see Aldao, 2013; Bonanno and Burton, 2013; Aldao et al., 2015; Gross, 2015).

The majority of past experimental paradigms have examined ER by focusing on the application or effectiveness of a single ER strategy. For example, several studies presented participants with emotion eliciting stimuli (i.e., pictures and film clips) and instructed them to implement a given strategy to regulate their affect (e.g., Ochsner et al., 2002; Sheppes et al., 2014). A central assumption of these paradigms is that participants will use one strategy (e.g., reappraisal) for the entire duration of the stimulus (on average, 2–5 min for film clips or 8 s for individual pictures). On the other hand, whether in the laboratory or in everyday life, people are typically not limited to one ER strategy, but rather, they may spontaneously employ a repertoire of multiple strategies to regulate emotions in response to the same events to ensure successful ER. To examine this possibility in the laboratory environment, Aldao and Nolen-Hoeksema (2013) identified the number of ER strategies endorsed by participants watching a film clip depicting amputations and reported that the majority of participants (65%) used multiple ER strategies to regulate their disgust. Similarly, in a set of four recent experiments examining uninstructed ER choice (Opitz et al., 2015) approximately 25%, of participants in all but one study reported using multiple ER strategies. These patterns of spontaneous ER use have also been reported outside of the laboratory. For example, in a study employing Ecological Momentary Assessment, Heiy and Cheavens (2014) found that participants used an average of seven ER strategies to regulate each instance of negative emotions and an average of eight ER strategies to regulate every instance of positive emotions.

One of the factors that determine which ER strategy will be employed depending on the situation is *context sensitivity*, which pertains to an individual’s ability to appraise situational demands and flexibly select the most adaptive ER strategy according to context. For example, a series of studies using a recall paradigm in which participants described past real-life situations found that three factors impacted ER strategy use: type of emotion (i.e., anger or sadness), level of intensity of the emotion (i.e., high or low), and specific life domain (i.e., achievement-related or social-related; Aldao and Nolen-Hoeksema, 2012; Dixon-Gordon et al., 2015a,b). In addition, the controllability of a situation has also been suggested as a contextual factor that influences the effectiveness or appropriateness of an ER strategy. In support of this proposal, a recent study using an ER task in which participants were instructed to reappraise sad film clips, showed that cognitive reappraisal ability (CRA; the ability to use reappraisal in response to emotional stimuli) was differentially effective depending on whether a stressor was controllable or uncontrollable (Troy et al., 2013). Specifically, in uncontrollable situations, higher CRA was associated with lower levels of depression, whereas in controllable situations, higher CRA was associated with heightened levels of depression. For example, a student responding to a failed exam could use reappraisal to tell herself that they “tried their hardest” which could be beneficial in the short-term; however, a problem-solving strategy (e.g., “let me find ways to do better next time”) would be more beneficial in the long term. Similarly, participants who regulated more flexibly by using more problem-focused coping in controllable situations and emotion-focused coping in uncontrollable situations were most successful in regulating their emotions both inside and outside of the laboratory (Cheng, 2001). Overall, these findings suggest that the ability to take into consideration contextual factors may be critical for choosing an appropriate and effective ER strategy. However, only a handful of studies have focused on the impact of individual differences on the expression of these effects.

Although research focusing on gender differences in ER flexibility is limited, Cheng (2001) examined possible gender effects in the flexible deployment of different coping strategies. The results demonstrated that women were more flexible in their use of problem-focused and emotion-focused coping strategies relative to men, a difference that was present both inside and outside of the laboratory. Further work specifically targeting gender differences in ER strategy use has employed self-report measures that included questions about what participants typically did to regulate their emotions (for a review see Tamres et al., 2002). These measures may tap into trait or habitual regulatory differences in that they require the participant to indicate what they would “generally do” or “usually do” across many different situations (for example, the “COPE” inventory; Carver et al., 1989). Nevertheless, these questionnaires inherently cannot take into consideration context sensitivity, as they do not distinguish among different situations. Even though these self-report questionnaires do not capture context sensitivity *per se*, a meta-analysis (Tamres et al., 2002) examined gender effects in ER across studies that used such self-report measures to capture ER strategy use in specific contexts (e.g., a relationship break-up, Choo et al., 1996; work stressors, Gianakos, 2000). The results of this meta-analysis revealed that women were significantly more likely than men to use eleven out of seventeen recorded strategies, across contexts. However, when the nature of the stressor was taken into account, men were more likely to vent in response to achievement and relationship stressors and more likely to use avoidance to cope with relationship stressors. Similarly, women were more likely to select isolation as a coping strategy in response to relationship stressors, whereas men were more likely to use the same strategy in response to others’ health stressors (Tamres et al., 2002). These findings highlight the importance of taking into consideration context sensitivity in ER research, especially in regards to gender differences.

### The Present Study

The aim of the present study was to examine potential gender differences on two components of ER flexibility, namely, context sensitivity and repertoire. To achieve this, we used a novel paradigm which exposed participants to three hypothetical real-world scenarios and subsequently asked them to free write about what actions they would take in response to each. In line with past research highlighting the importance of varying the nature (Tamres et al., 2002), intensity (Dixon-Gordon et al., 2015a), and controllability of a stressor (Troy et al., 2013; Ford and Gross, 2019), and of examining a larger number of ER strategies (Heiy and Cheavens, 2014; Dixon-Gordon et al., 2015b), here we employed three scenarios that varied regarding the area of life they pertained to (academic, romantic, and health), their intensity (high vs. low), and level of controllability of the event taking place (high vs. low). Based on past literature, we predicted that, across scenarios, women would have a larger repertoire and be more flexible in the implementation of different ER strategies than men.

## Materials and Methods

### Participants

Participants were 112 native English speakers between the ages of 18 and 31 (*N* = 112; mean age = 19, *SD* = 1.51; 62 [55%] female). A majority of the sample identified as Caucasian (*N* = 93, 83%), and the rest identified as: Asian *n* = 9, Black *n* = 5, more than one *n* = 3, and other *n* = 1. The participants were recruited via SONA through the introductory psychology pool at a large mid-western university and they were not screened based on gender, race, or handedness. The study was approved by the local institutional review board; participants were required to provide consent and were debriefed and given course credit for their time.

### Materials

#### Emotion Eliciting Scenarios

Participants were instructed to read three hypothetical scenarios meant to elicit negative emotions. These scenarios came from three separate contexts or life domains: academic (achievement related), romantic relationship, and health. The academic scenario was: “*Imagine that you just found out that you failed a really important test and might have to retake the course because of it. You prepared a lot for the test and are disappointed that you did not do better. This is one of your least favorite courses and you often complain about the instructor to your friends*.” The romantic scenario was: “*Imagine that you just found out that your partner (boyfriend/girlfriend) has been cheating on you. Your partner has told you that this has been going on for a few months. Your partner tells you that he/she felt like something was lacking in your relationship and that they feel their relationship with this other person is more fulfilling. Your partner tells you that they are sorry, but that the two of you need to break up*.” The health scenario was: “*Imagine that you have not been feeling very well and when you went to the doctor he/she ordered several tests and one of them came back positive. You are going to need to have an operation within the next couple of weeks. Your doctor assures you that it is a simple procedure, but that you will have to stay in the hospital for a couple of days afterwards. You do some research online and find out that while the operation is usually successful with no problems, there have been several people who’ve had serious complications from the operation*.”

#### Development of Scenarios

##### Stimuli development

We obtained controllability and intensity ratings for each scenario through the recruitment of a separate sample of undergraduate students (*N* = 22). Repeated measures ANOVAs were used to test for group differences across the three scenarios. Level of controllability was significantly higher for the academic scenario than both the health scenario, *F*(2,27) = 9.55, *p* = 0.005, $\eta_{p}^{2}$ = 0.26, and the romantic scenario *F*(2,27) = 9.11, *p* < 0.001, $\eta_{p}^{2}$ = 0.18. The romantic and health scenarios were equally low on controllability, *F*(2,27) = 2.84, *p* = 0.103, $\eta_{p}^{2}$ = 0.09. There were no significant gender differences in ratings of controllability among scenarios (all *p*s > 0.05).

Negative emotional intensity was significantly higher in the romantic scenario relative to both the academic, *F*(2,27) = 20.42, *p* < 0.001, $\eta_{p}^{2}$ = 0.01, and health, *F*(2,27) = 23.4, *p* < 0.001, $\eta_{p}^{2}$ = 0.46, scenarios. The academic and health scenarios were equally low on intensity (*p* > 0.05). However, there were significant gender differences in ratings of emotional intensity. Females viewed both the romantic [*t*(26) = 2.18, *p* = 0.038, *d* = 0.78], and health [*t*(26) = 2.22, *p* = 0.035, *d* = 0.89] scenarios as being more intense than men.

Overall the results of this norming study confirmed the variability of our scenarios and showed that the academic scenario was higher on controllability and lower on intensity; the romantic scenario was lower on controllability and higher in intensity; and the health scenario was lower on both controllability and intensity. We note that, when taking gender differences into account, women view both the romantic and health scenarios as significantly more intense than men.

#### Measures of Affect and Mood

##### PANAS (Watson et al., 1988)

The PANAS is a self-report measure designed to assess both positive and negative affect. The PANAS consists of 20 adjectives pertaining to negative affect (i.e., distressed or nervous) and positive affect (i.e., excited or proud), with ten items for each subscale. Items are rated on a five-point Likert scale: 1 = “Very slightly or not at all” to 5 = “Extremely.” The subscales are obtained by taking the average of each item within that subscale.

#### Beck Depression Inventory – Second Edition (BDI-II; Beck and Steer, 1984)

The BDI-II is a self-report measure designed to assess depressive symptomatology. The BDI-II provides one overall score (range 0–63). A score of ≤13, 14–19, 20–28, and ≥29 represent minimal, moderate, and severe depressive symptoms, respectively.

### Procedure

Following informed consent, the participants filled out the PANAS questionnaire to measure current mood and the BDI. Participants with scores > 10 on the BDI were not included in the study; thus, the sample consisted of only non-depressed subjects. The participant then began the ER portion of the study, during which participants read the three hypothetical situations that were meant to elicit negative emotions in the following order: academic, romantic, and health. The scenarios were shown in the same order for each participant because we wanted to ensure that we could compare strategies used for each participant taking into consideration the order in which the scenarios were shown (e.g., that the romantic strategies selected could have been influenced by the academic scenario, but that would be constant for all participants). Immediately following each scenario they were asked to free write for 5 min about what they would do or say if the event had happened in their life. They then ranked 12 ER strategies based on the likelihood they would use each one. Immediately following the ranking of strategies, the participant was shown each of the 12 strategies separately and was asked to rate the extent to which they would use each one on a scale from 0 “Not at all” to 4 “A lot.” The rankings and ratings were included to ensure that each participant was able to indicate which strategies they would use. For example, some participants may not have been strong writers, which could have influenced their free writing responses and not fully represented what they would do in each situation. To ensure that participants had a good understanding of each strategy, we provided them with a brief example of each that corresponded to the way they are commonly conceptualized in the literature. The examples shown to participants can be found in Table 1. After the participant completed the three scenarios, they filled out the final PANAS questionnaire to measure their mood. The participant then completed several self-report measures. Lastly, subjects were given a debriefing form and a short summary of the experiment verbally.

**Table 1:** Summary of coding scheme: descriptions of strategies.

Strategy (category)	Description	References
Rumination	Thinking all of the time about the feelings and thoughts associated with the negative event.	Nolen-Hoeksema et al., 1994
Reappraisal	Cognitively transforming the situation to alter its emotional impact; can involve generating positive interpretations of the event.	Gross, 1998; Aldao et al., 2010
Suppression	Consciously trying to stop thinking about a particular thought or situation.	Wegner et al., 1987
Expressive Suppression	Consciously inhibiting the expression of emotion.	Bonanno and Burton, 2013
Problem solving	Attempting to change a stressful situation; thinking about what steps to take to deal with the event.	Carver et al., 1989; Aldao et al., 2010
Acceptance	Resigning to what has happened or accepting the reality of the situation; acceptance of thoughts and feelings.	Carver et al., 1989; Hayes et al., 2004
Self-blame	Blaming yourself for what you have experienced.	Garnefski et al., 2001
Other-blame	Putting the blame for what you have experienced on others.	Garnefski et al., 2001
Perspective	Playing down the seriousness of the event or emphasizing its relativity when compared to other events.	Garnefski et al., 2001
Denial	Refusing to believe that the stressor exists or trying to act as though the stressor is not real.	Carver et al., 1989
Behavior	Engaging in a behavior (i.e., reading, going for a walk, hanging out with a friend) to take your mind off a situation or distract yourself.	Carver et al., 1989
Impulsive behavior	Engaging in an impulsive behavior (i.e., drinking, drug use, and binge eating) to distract or suppress emotional states.	Carver et al., 1989
Social support	Seeking social support for instrumental reasons (i.e., seeking advice or information) or seeking social support for emotional reasons (i.e., getting sympathy or understanding.)	Carver et al., 1989
Catastrophize	Expecting or worrying about major negative consequences from a situation, even one of minor importance.	Rosenstiel and Keefe, 1983
Emotion expression	How one conveys emotional experience through both verbal and non-verbal behaviors.	Gross, 1998
Religion	Using religion or spiritual beliefs/resources to cope.	Carver et al., 1989
Emotion label	Labeling the emotion(s) you would feel.	n/a
Miscellaneous	When a sentence does not relate to any of the strategies.	n/a


### Data Preparation

#### Coding Procedure

The participants’ free-writing responses were coded for specific ER strategy usage following a coding scheme developed specifically for this study. To compose this scheme, we compiled and synthesized definitions and examples of ER strategies from the literature on affective regulation and coping. The coding scheme included sixteen strategies, emotion labels, and a miscellaneous category for instances in which a sentence did not contain an ER strategy. The complete coding scheme is presented in Table 1 (description) and Table 2 (examples). Each participant’s response for each scenario was first segmented into sentences. Each sentence was coded separately. A given sentence could contain multiple ER strategies. A single strategy could also be used more than once for each scenario (e.g., a participant endorsed three different ways to problem solve). All coding was performed by three independent coders, each blind to identifying information on each subject. Disagreements among raters were resolved in conference. Cohen’s Kappa was used to determine the interrater reliability for the 1,667 total coded statements, *κ* = 0.75 (95% CI, 0.56 to 0.91), *p* < 0.001, which is considered to reflect substantial interrater agreement (Landis and Koch, 1977).

**Table 2:** Summary of coding scheme: examples of strategies.

Strategy (category)	Example	References
Rumination	“I would think over and over again about the situation and my feelings”	Treynor et al., 2003
Reappraisal	“I would think about the situation differently in order to change how I felt”	Aldao and Nolen-Hoeksema, 2012
Suppression	“I would try not to think about the consequences”	n/a
Expressive Suppression	“I would control my emotions by not showing them”	Gross and John, 2003
Problem solving	“I would come up with ideas of how to change the situation or fix the problem”	Aldao and Nolen-Hoeksema, 2012
Acceptance	“I would accept or allow my feelings”	Aldao and Nolen-Hoeksema, 2012
Self-blame	“I would think that basically the cause must lie in myself”	Garnefski et al., 2001
Other-blame	“I would feel that others were responsible for what happened”	Garnefski et al., 2001
Perspective	“I would tell myself that there are worse things in life”	Garnefski et al., 2001
Denial	“I would just act like the situation had never happened at all”	Carver et al., 1989
Behavior	“I would turn to work or other activities to take my mind off of things”	Carver et al., 1989
Impulsive behavior	“I would drink alcohol/binge on food”	Carver et al., 1989
Social support	“I would try to get advice from someone about what to do”	Carver et al., 1989
Catastrophize	“I would think that it’s terrible and it’s never going to get any better”	Rosenstiel and Keefe, 1983
Emotion expression	“I would cry”	n/a
Religion	“I would pray”	Carver et al., 1989
Emotion label	“I would feel sad”	n/a
Miscellaneous	No strategy endorsed.	n/a


#### Variable Creation

Beyond the variation in specific strategy use across contexts, we calculated a number of additional summary variables that would provide overall measures of ER flexibility (partly based on recommendations from Aldao, 2013), as follows:

##### Regulatory effort

For each scenario, the participant received a score reflecting how many times they tried to regulate their emotions. This was calculated by totaling the sum of strategies endorsed for that scenario. If the same strategy was endorsed multiple times in the scenario, each of the instances of regulation counted toward this score. For example, if a participant endorsed two instances of reappraisal, three instances of problem solving, and one instance of perspective taking they were given a score of six for regulatory effort.

##### Total distinct strategies

For each scenario the participant received a score reflecting how many distinct strategies they used to regulate their emotions. This was calculated by counting the sum of distinct strategies endorsed for that scenario. Unlike *Regulatory Effort*, if the same strategy was endorsed multiple times in the scenario, it only contributed a score of one to the total distinct strategies score. Using the example above, if a participant endorsed two instances of reappraisal, three instances of problem solving, and one instance of perspective taking the participant was given a score of three (instead of six) because only three distinct strategies were used across the scenario, regardless of how many times each was endorsed.

##### Flexibility

For the romantic and health scenarios the participant received a score capturing the total number of novel strategies used in that scenario. Flexibility was, thus, operationalized as how many distinct *and* unique (i.e., not having been used before) strategies the participant endorsed for that scenario. For the romantic scenario, this was calculated by taking the total distinct strategy scores for the academic and romantic scenarios and only counting the strategies endorsed in the romantic scenario that were *not* endorsed in the academic scenario. For the health scenario, this was calculated by taking the total distinct strategy scores for all three scenarios and only counting the strategies endorsed in the health scenario that were not endorsed in either the academic or romantic scenarios.

##### Repertoire

Each participant was given a repertoire score that represented the total number of distinct strategies used across the three scenarios. This measure pertained to how many individual strategies a participant employed, overall (i.e., trait ER strategies). This was calculated by summing the total distinct strategies score for the academic scenario and the total novel strategies scores for the romantic and health scenarios.

##### Total strategies

Each participant was given a total strategies score that represented the total instances of regulation across the three scenarios. We conceptualized this as how many times the individual attempted to regulate their emotions across the three scenarios. This was calculated by summing the total regulatory effort scores for each scenario. Strategies that were used more than once both within and across the three scenarios would be included in this total score.

## Results

### Positive and Negative Affect

Prior to the ER there were no significant differences for either negative [*F*(1,110) = 1.24, *p* = 0.27, η^2^= 0.01] or positive affect [*F*(1,110) = 1.09, *p* = 0.30, η^2^= 0.01] between males and females. Similarly, following ER there were no significant differences for either negative [*F*(1,110) = 0.01, *p* = 0.92, η^2^ < 0.001] or positive affect [*F*(1,110) = 0.02, *p* = 0.90, η^2^ < 0.001] between males and females. Thus, any differences between males and females are not likely attributed to *a priori* differences in current mood or differential effects of the task on current mood between the two groups.

### Gender Effects on ER Flexibility in Free Writing

#### Overall Differences in ER Flexibility

**FIGURE 1:**
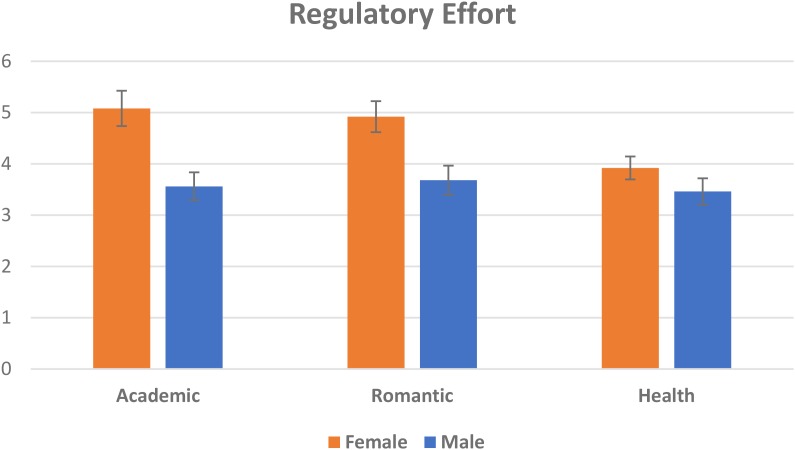
Means and standard deviations for gender differences in regulatory effort within each scenario. Error bars represent the standard error of the means.

**FIGURE 2:**
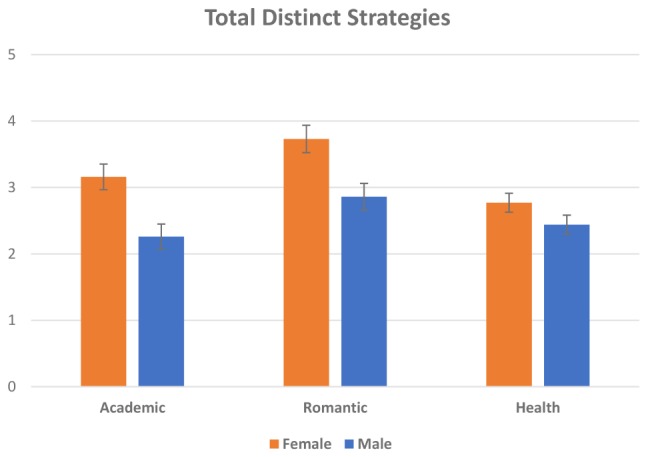
Means and standard deviations for gender differences in total distinct strategies within each scenario. Error bars represent the standard error of the means.

To test for the influence of gender on ER flexibility we first ran two repeated measures mixed ANOVAs with the Total Strategies (i.e., the sum of Regulatory Effort scores across scenarios) and the Total Distinct Strategies in each scenario as the within-subjects factors and gender as the between-subjects factor. For both factors, the results revealed significant interaction effects between context and gender, with females exhibiting higher total strategies *F*(1.84,202.40) = 3.51, *p* = 0.03 = η^2^ = 0.09 (Figure 1) and using more total distinct strategies than men, *F*(1.86,204.96) = 4.62, *p* = 0.03, η^2^ = 0.15 (Figure 2), for each of the three scenarios. We then ran two independent samples *t*-tests to examine Flexibility [i.e., the distinct *and* unique (i.e., not having been used before) strategies the participant endorsed for the romantic and health scenarios]. Women had higher Flexibility for the romantic scenario *t*(110) = 2.01, *p* = 0.035, *d* = 0.38, however, there were no significant gender differences in the health scenario (*p* > 0.05) for this measure. Finally, we ran two independent samples *t*-tests for Repertoire and Total strategies. Women had a significantly larger repertoire than men, *t*(110) = 3.86, *p* < 0.001, *d* = 0.74 and used a significantly larger amount of total instances of regulation, overall, than men *t*(110) = 3.60, *p* < 0.001 (see Table 3 for descriptive statistics).

**Table 3:** Means and standard deviations of gender differences in emotion regulation flexibility variables.

		*M* (*SD*)		
Variable	Scenario	Female	Male	*p*-value
Regulatory effort	Academic	5.08 (2.71)	3.56 (1.93)	0.001
	Romantic	4.92 (2.38)	3.68 (2.02)	0.004
	Health	3.92 (1.77)	3.46 (1.82)	*NS*
Total distinct strategies	Academic	3.16 (1.52)	2.26 (1.34)	0.001
	Romantic	3.73 (1.62)	2.87 (1.43)	0.004
	Health	2.77 (1.22)	2.44 (1.01)	*NS*
Flexibility	Romantic	2.58 (1.35)	2.08 (1.26)	0.04
	Health	1.15 (0.88)	1.08 (1.01)	*NS*
Repertoire	Sum	6.89 (1.94)	5.40 (2.07)	<0.001


*M, mean; SD, standard deviation; NS, not significant.*

#### Differences by ER Strategy Within Scenarios

To examine gender differences in specific strategy usage we first ran independent samples *t*-tests for each individual strategy within each scenario. For the academic scenario, each of the following strategies was used significantly more by female than male participants: problem solving *t*(110) = 2.11, *p* = 0.04, *d* = 0.40, social support *t*(110) = 2.26, *p* = 0.03, *d* = 0.43, and emotion expression *t*(110) = 3.47, *p* = 0.001, *d* = 0.77. For the romantic scenario, each of the following strategies was used significantly more by females than males: self-blame *t*(110) = 2.77, *p* = 0.007, *d* = 0.53, social support *t*(110) = 2.46, *p* = 0.02, *d* = 0.50, and emotion expression *t*(110) = 2.18, *p* = 0.03, *d* = 0.43. For the health scenario, acceptance was endorsed significantly more by males than females *t*(110) = 2.16, *p* = 0.03, *d* = 0.41 (see Table 4 for descriptive statistics).

**Table 4:** Means and standard deviations for gender differences in individual strategy use within each scenario.

			*M (SD)*		
Scenario	Method	Strategy	Female	Male	*p*-value
Academic	FW	Problem solving	2 (1.52)	1.46 (1.11)	0.04
	FW	Social support	0.5 (0.59)	0.26 (0.53)	0.03
	FW	Emotion expression	0.29 (0.54)	0 (0)	0.001
	SR	Acceptance	0.85 (0.35)	0.98 (0.14)	0.01
	SR	Denial	0.26 (0.44)	0.54 (0.50)	0.002
	SR	Suppression	0.74 (0.44)	0.92 (0.27)	0.01
	SR	Social support	0.97 (0.84)	0.84 (0.37)	0.03
Romantic	FW	Self-blame	0.63 (0.79)	0.28 (0.54)	0.01
	FW	Social support	0.61 (0.91)	0.28 (0.50)	0.02
	FW	Emotion expression	0.42 (0.71)	0.18 (0.44)	0.03
	SR	Suppression	0.74 (0.44)	0.92 (0.27)	0.01
Health	FW	Acceptance	0.65 (0.81)	1.00 (0.93)	0.03
	SR	Reappraisal	0.92 (0.28)	0.78 (0.42)	0.04
					


*FW, free-writing; SR, self-reported ratings; M, mean; SD, standard deviation.*

#### Differences by ER Strategy Across Scenarios

For each strategy a total score was calculated by adding the instances the participant made use of the strategy across the three scenarios. These total scores followed a similar pattern as the individual strategy use within scenarios, with females endorsing significantly higher strategy use than males for most strategies and males endorsing only one strategy significantly more than females. Women used the following strategies significantly more than men: problem solving *t*(110) = 2.09, *p* = 0.04 *d* = 0.40, self-blame *t*(110) = 2.78, *p* = 0.006, *d* = 0.51, social support *t*(110) = 2.63, *p* = 0.01, *d* = 0.51, and emotion expression *t*(110) = 3.65, *p* < 0.001, *d* = 0.75. As with the health scenario, males endorsed acceptance more than females *t*(110) = 2.68, *p* = 0.009, *d* = 0.56 (see Table 5 for descriptive statistics).

**Table 5:** Means and standard deviations for gender differences in total individual strategy use.

		*M (SD)*		
Strategy	Method	Female	Male	*p*-value
Problem solving	FW	2.94 (2.21)	2.14 (1.70)	0.04
Self-blame	FW	1.26 (1.41)	0.62 (1.01)	0.01
Social support	FW	1.71 (1.73)	1.00 (1.11)	0.01
Emotion expression	FW	0.74 (0.97)	0.22 (0.51)	<0.001
Acceptance	FW	1.00 (1.11)	1.70 (1.40)	0.01
Social support	SR	2.90 (0.35)	2.60 (0.81)	0.04
Suppression	SR	2.37 (0.91)	2.74 (0.66)	0.02


*FW, free-writing; SR, self-reported ratings; M, mean; SD, standard deviation*.

### Gender Effects on ER Flexibility in Self-Report Ratings

The self-report ratings of strategy usage provided by participants were partially consistent with the free-writing data. As discussed previously, these ratings provide important ancillary information on ER ability beyond the free writing data, in that some participants may not have been strong writers or may have had trouble thinking about what they would do. Similar to Aldao and Nolen-Hoeksema (2013), we dichotomized the strategy ratings so that if a participant endorsed a strategy “A little,” “Somewhat,” or “A lot” we considered that strategy to have been used. If the participant endorsed a strategy “Not at all,” we assumed that they would not have used the strategy. In line with the free-writing results, the self-report rating analysis showed that females endorsed more social support in the academic scenario *t*(110) = 2.24, *p* = 0.03, *d* = 0.45, the romantic scenario *t*(110) = 2.14 *p* = 0.04, *d* = 0.42, and overall *t*(110) = 2.48, *p* = 0.02, *d* = 0.49. In deviation from the free-writing analysis, according to the self-report ratings, men used significantly more than women suppression *t*(110) = -2.49, *p* = 0.01, *d* = 0.48, acceptance, *t*(110) = -2.54, *p* = 0.01, *d* = 0.48, and denial *t*(110) = -3.11, *p* = 0.002, *d* = 0.59, for the academic scenario; and suppression *t*(110) = -2.49, *p* = 0.014, *d* = 0.48 for the romantic scenario, as well as total suppression *t*(110) = -2.48, *p* = 0.015, *d* = 0.46 across scenarios. Women only endorsed reappraisal in the health scenario *t*(110) = 0.78, *p* = 0.035, *d* = 0.39 significantly more than men (see Tables 4, 5 for descriptive statistics).

### Summary

Gender had an impact on both individual strategy use and ER flexibility. Overall, when there were gender differences, women tended to use ER strategies more often than men and in a more flexible manner. The only strategies that men used significantly more than women was acceptance according to the free-writing and suppression and denial according to the self-report ratings.

## Discussion

As researchers argue for a personalized science of ER, acknowledging individual differences is critical (Schmeichel and Tang, 2015; Doré et al., 2016). The high prevalence of gender differences in psychopathologies that include ER deficits points toward gender as an important such individual differences factor (Hyde et al., 2008; Nolen-Hoeksema, 2012). Gender differences have been found in many ER processes, including specific ER strategies and neural systems involved in ER. However, gender differences in ER flexibility remain a largely unexplored topic. This study is among the first to examine directly gender variation in the employment of these processes.

The results of our experiment point toward possible gender differences in both specific strategy usage and the flexibility with which these strategies are implemented. In line with past research (e.g., Nolen-Hoeksema and Jackson, 2001; Tamres et al., 2002; Nolen-Hoeksema and Aldao, 2011; Kwon et al., 2013), women had a significantly larger repertoire than men, suggesting that women may have access to a greater number of strategies than men depending on context. Females also showed higher levels of regulatory effort both within each individual scenario and across all three scenarios. One possible interpretation of these findings would suggest that women put more effort into their affect regulation and try more ER strategies than men. On the other hand, this increase in effort may not necessarily be effective for ER. In line with this proposal, McRae et al. (2008) found that during a reappraisal task, men showed less activity in prefrontal cortex regions and greater decreases in amygdala activity than women. These findings were interpreted as reflecting more automatic and effective ER in men, although the results have not been consistently replicated (Domes et al., 2010). Moreover, increased activation in a brain region does not indicate with certainty more efficient cognitive or affective processing (Poldrack, 2010). Nevertheless, we note that quantitative differences in the use of ER strategies do not necessarily imply qualitative differences in ER effectiveness and implementation across contexts.

More specifically related to flexibility, women used significantly higher levels of distinct strategies across the three scenarios. Overall these results suggest that women tended to use most ER strategies more often than men and in a more flexible manner. These findings are in line with the results of the meta-analysis by Tamres et al. (2002) who showed that women used the vast majority of strategies across the three scenarios significantly more than men, including problem-solving, self-blame, social support, and emotion expression. It has been suggested that women tend to use self-blame more than men because they are more likely to view their emotions as the result of something internal rather than something specific to that situation, a tendency that may also lead to a woman feeling her emotions are out of her control (Nolen-Hoeksema and Jackson, 2001; Nolen-Hoeksema, 2012). We did not specifically ask participants how controllable they felt the situation was; however, the results of our norming study during the development of our scenario stimuli indicated that the romantic scenario was perceived as low on controllability relative to the other scenarios. Thus, it is possible that in a situation that is perceived as uncontrollable, women are more likely to blame themselves for whatever has happened. Finally, across scenarios, women used social support significantly more than men both in the free-writing data and self-report ratings. This is consistent with the findings by Tamres et al. (2002), according to which women were more likely than men to seek both emotional and instrumental support consistently across studies and contexts.

This pattern of results held when examining strategy usage for each individual scenario, with the exception of the health scenario, which was the only scenario in which men used acceptance more than women. This scenario was the only one rated low in both controllability and intensity in our norming study. Thus, one potential explanation for these findings is that men are only more likely to use acceptance as an ER strategy in situations that are both low on controllability and intensity.

In contrast to the free-writing analysis, an examination of the self-report data revealed that men used suppression significantly more than women in both the academic and romantic scenarios, denial more in the academic scenario, and more suppression, overall, across scenarios. Interestingly, these results differed somewhat from those reported by Tamres et al. (2002). According to their findings, men use more avoidant and withdrawal strategies in situations they potentially view as less controllable (e.g., pertaining to romantic relationships or someone else’s health), whereas our study suggests that men may use these strategies in both controllable and uncontrollable situations. Although these two scenarios were similar in their level of intensity overall (across genders), men tended to view the romantic scenario as significantly less intense than women. This suggests that men may use such avoidant strategies more in situations they have a lesser emotional response to. Nevertheless, the employment of such strategies may not necessarily result in effective ER. Although we did not measure strategy effectiveness in the present design, other studies have compared variations in strategy effectiveness. For example, Sheppes et al. (2014; see also Sheppes et al., 2011) showed that distraction was a more effective ER strategy specifically for higher intensity negative picture stimuli across genders. We note that our participants did not directly provide, intensity and controllability ratings for each scenario, which were only collected during stimuli norming. The inclusion of such ratings in future studies, may elicit a different profile of results further elucidating the impact of the participant’s perception of a situation on ER strategy use.

From a methodological standpoint, the present study constitutes a possible departure from the usual way of evaluating the implementation of ER strategies through the administration of self-report questionnaires that tap on trait level processes. Instead, here, we asked individuals to identify specific ER strategies they would employ in response to specific emotional events as captured in real-world hypothetical scenarios using free writing. This unique methodological approach allowed us to examine, for the first time, contextual variations in the implementation of ER strategies, a critical area for our understanding of ER processes that remains largely understudied.

Despite its unique contributions, our study was unable to examine whether the participants’ regulation attempts were effective or not. Future research should examine the effectiveness of a strategy (or strategies) in different contexts, as well as an individual’s responsiveness to feedback in switching between strategies or moving away from ineffective strategies. Similarly, although three scenarios is a step in the right direction in examining context sensitivity, future research should include a wider range of contextual factors. For example, the specific emotion elicited by a given situation may impact significantly ER strategy usage (e.g., Dixon-Gordon et al., 2015a). Although the present study did not ask participants what emotion(s) they would feel in response to the scenario, future research should ask this question directly. As discussed previously, we only obtained ratings of controllability and intensity for the scenarios during stimulus development. In the future it may be beneficial to ask participants completing different ER tasks about these two factors to more directly examine their impact on strategy use. Furthermore, to control for variability in practice effects across the scenarios, each participant was shown the three scenarios in the same order; this introduces the possibility that the responses for the second and third scenarios could have been influenced by the previous scenario. Future research might benefit from randomizing the order in which participants are shown each context, although we note that randomization would not fully eliminate such crossover effects. A factor that may have influenced the pattern of results pertains to possible gender differences in theory of mind (ToM) abilities (e.g., Rowland et al., 2013; Adenzato et al., 2017), which could have interacted with ER regarding the ease in which participants were able to see themselves in the three scenarios used. Future work in this field should incorporate ToM measures to examine this possibility. Finally, although equated for gender, our sample was restricted to college-aged students in a United States institution; thus, the generalizability of the results is necessarily limited to this population and may not extend to other ages or cultures.

In much previous research on ER, gender has been a neglected factor. The findings of this study highlight the importance of considering gender differences in ER research. Many results pertaining to ER processes are based on findings from only one gender or mixed samples where gender differences are not analyzed because of unequal or small sample sizes. This poses a critical issue as some researchers argue that in a context where behavioral and neural gender differences have been found, it is imperative that we examine and address potential gender differences for valid and generalizable results. A more detailed and complete understanding of gender differences in ER processes is needed to understand more fully both the nuances of ER processes and to develop effective treatments for the multitude of psychopathologies that have ER deficits at their core.

## Ethics Statement

This study was carried out in accordance with the recommendations of the Human Subjects Research guidelines of the University of Kansas Institutional Review Board with written informed consent from all subjects. All subjects gave written informed consent in accordance with the Declaration of Helsinki. The protocol was approved by the University of Kansas Institutional Review Board.

## Author Contributions

EC and KG conceptualized and designed the research. KG prepared and conducted the experiments and collected the data. KG prepared the data for analysis. EC and KG analyzed the data and wrote the manuscript.

## Conflict of Interest Statement

The authors declare that the research was conducted in the absence of any commercial or financial relationships that could be construed as a potential conflict of interest.
